# NovelSNPer: A Fast Tool for the Identification and Characterization of Novel SNPs and InDels

**DOI:** 10.1155/2011/657341

**Published:** 2011-10-31

**Authors:** Jens Aßmus, Armin O. Schmitt, Ralf H. Bortfeldt, Gudrun A. Brockmann

**Affiliations:** ^1^Department of Crop and Animal Sciences, Humboldt-University of Berlin, Invalidenstraße 42, 10115 Berlin, Germany; ^2^Faculty of Science and Technology, Free University of Bozen, Piazza Università 5, 39100 Bozen, Italy

## Abstract

Typically, next-generation resequencing projects produce large lists of variants. NovelSNPer is a software tool that permits fast and efficient processing of such output lists. In a first step, NovelSNPer determines if a variant represents a known variant or a previously unknown variant. In a second step, each variant is classified into one of 15 SNP classes or 19 InDel classes. Beside the classes used by Ensembl, we introduce POTENTIAL_START_GAINED and START_LOST as new functional classes and present a classification scheme for InDels. NovelSNPer is based upon the gene structure information stored in Ensembl. It processes two million SNPs in six hours. The tool can be used online or downloaded.

## 1. Background

One of the main goals of resequencing projects is to detect genomic variation between an individual of interest and the reference genome that is stored in public databases. Typically, the whole genome or a section of the genome sequence encompassing the genomic region of interest is chosen as reference sequence for the resequencing project. The DNA sample is manually enriched for nonrepetitive sequence in the target genomic region using capture oligomers before performing the sequencing reactions. Depending on the technology, reads of length in the range between a couple of dozens and a few hundred nucleotides result. One sequencing reaction typically yields in the order of dozens of millions of reads.

Thus far, several fast and robust programs are available to assemble the reads from an NGS experiment, to compare the resulting consensus sequence with the reference sequence and to extract genomic variations [1–4]. However, only few tools are available to date that offer support for the further characterization of identified variations. SNP annotation is a largely standardized process performed in software pipelines at the large genome centers [5, 6]. Previously, Ensembl made its SNP annotation algorithm accessible via the tool “Variant Effect Predictor” [7]. SNP annotation in more specific contexts is furthermore performed by a series of other software tools [8–12]. However, with the rapid spreading of high-throughput sequencing machines, there is a growing need for high-quality assembly and annotation tools to cope efficiently with the massive amount of sequencing data in respect to annotation and speed data analysis and to extend the analysis to indels.

The motivation to develop NovelSNPer was to offer an easy-to-use and fast tool that allows the distinction between variations that are already classified as reference variations (e.g., SNPs that were assigned an rs-number and that were stored in dbSNP [13]) and previously unknown variations which were discovered in the sequencing data and to characterize the variations. Other tools with a similar scope that were recently developed as, for example, SNPnexus [14] or VarySysDB [15] harness a variety of external data sources to characterize polymorphisms, but are based on static datasets restricted to human and offer only limited batch processing capabilities.

Our classification scheme comprises twenty-one variation classes that were defined largely analogously to those applied by Ensembl [16] (for further detail see Table 1). The usage of NovelSNPer is not restricted to humans. NovelSNPer is applicable to lists of variations deduced from resequencing data of all species whose genome sequence is stored in the Ensembl database. The only prerequisite is that the resequencing data was assembled to a genomic reference sequence which is stored in the Ensembl database.

## 2. Implementation

### 2.1. Input

NovelSNPer reads in an ASCII table (text file) that contains the information on all nucleotide polymorphisms between the derived sequence and the reference sequence in six columns: Name, Chromosome, RelStart, RelEnd, AltAlleles, and RefAllele, where Name represents an identifier for the variation chosen by the user, Chromosome is the chromosome number the variation was found on, RelStart and RelEnd are the position (start and end) of the variation in the reference sequence in base pairs (relative position), AltAlleles contains the nucleotide found at that position in the sequence data, and RefAllele is the nucleotide at that position in the reference sequence. In case of heterozygosity both alleles are specified in AltAlleles separated by a slash or in IUPAC code. Alternatively NovelSNPer reads a common variant reporting format (VCF or Personal Genome SNP format [17, 18]). The start position of the reference sequence relative to the chromosome sequence and the species must be specified as parameters on the command line upon execution of NovelSNPer.

There are three modes to use NovelSNPer.

- NovelSNPer uses the gene annotations from the Ensembl database to characterize the variations of the input file.

(ii) NovelSNPer uses the gene annotations from a local gene file to characterize the variations. The formats GFF and BED are supported [19, 20].

(iii) A genome annotation of a desired species from Ensembl is downloaded and saved at the home desktop by NovelSNPer.

An example for an input file is shown in Table 2. The command line syntax is NovelSNPer.pl-species human Input.txt.

**Figure 1:**
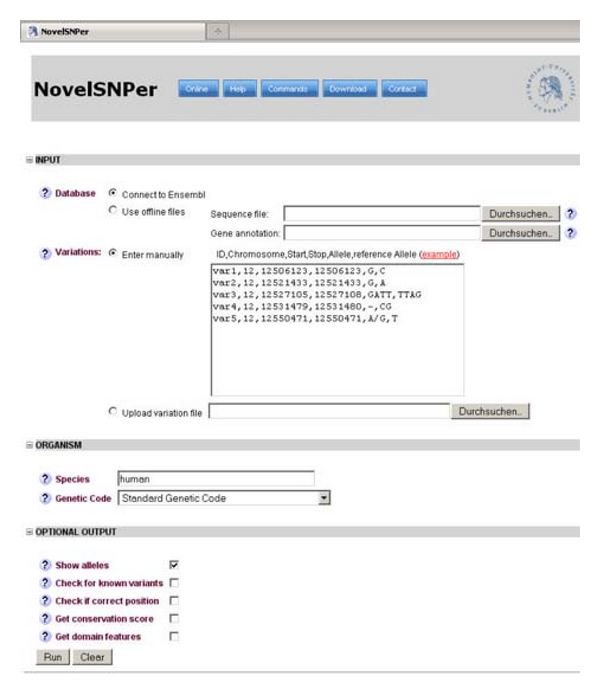
Web interface of NovelSNPer. Input file, species, genetic code, and other options can be entered into a graphical user interface at http://www2.hu-berlin.de/wikizbnutztier/software/NovelSNPer/.

Alternatively, the website can be used to evaluate the input file as shown in Figure 1. 

### 2.2. Output

 As output of a NovelSNPer run two tables are produced each in text and HTML format: one table provides a basic summary of the results for each variation, the other provides more detailed information about each transcript that is affected by a variation. The basic table contains the following data: Name, Chromosome, RelPos, ChromPos, FuncClass, NearestGene, Distance, Status, and ID. The meaning of the variables is given in Section 3. This table has the same number of entries as the input table and serves the scientists, who are interested in obtaining a quick overview about the variations identified in the resequencing data. The detailed table contains these variables: Name, Chromosome, RelPos, ChromPos, AltAllele1, AltAllele2, AltAllele3, RefAllele, Codon, AminoAcid, RefAminoAcid, FuncClass, Transcript, Strand, ID, ConsScore, and ProteinDomain. It serves the scientist, who is interested in performing a deeper analysis of individual variations above all by providing the transcript ID in which a variation was assigned a specific functional class. In the following, we will describe the variables of the detailed table that were not yet described earlier: The columns AltAllele1, AltAllele2, and AltAllele3 host the alleles that were found in the sequencing data. In case of homozygosity, only AltAllele1 will have an entry, the other two will be set to NA. In case of heterozygosity, both AltAllele1 and AltAllele2 will have an entry, but AltAllele3 will be set to NA. We provide the column AltAllele3 to account for cases of variations with three alleles that could be produced, for example, if the sequencing experiment was performed with a pool of DNA or in case of ambiguous variation calling. RefAllele is the nucleotide that is found in the reference sequence at the position of the variation. Codon, AminoAcid, and RefAminoAcid are only given in case the variation is in a coding region, otherwise NA is given. The nucleotide at the variable position in the codon is coded as IUPAC symbol. The variable Strand denotes whether the gene associated with a variation is annotated on the plus or minus strand of the reference genome. The user can choose to save the used Ensembl version and Assembly version in the file.

The tables in text format can be further processed in a statistical analysis program like R [21] or with a spread sheet like Excel. In the HTML table IDs, transcripts, genes, and protein domains are linked with Ensembl allowing easy access to further data. Furthermore, a report is produced containing key statistical figures like the number of variations processed, a listing of the number of variations in each functional class and the number of cases, in which the reference nucleotide that was specified in the input file did not coincide with the nucleotide of the reference sequence.

An example for the two output files is shown in Tables 3 and 4. Additionally the output can be converted into the GFF format and then be visualized using *Artemis* [22] or the *Integrated Genome Browser* [23].

### 2.3. Algorithm and Working Process

First, NovelSNPer connects to the Ensembl database and downloads the strand and the position of all transcripts and exons. Alternatively, NovelSNPer reads a GFF-file where the gene positions are annotated. Then NovelSNPer creates a hash function, which is referenced by the transcript IDs. The hash values are a list containing start, end, coding start, coding end, strand, and a hash function. This second hash function is referenced by the exons of the transcripts and its hash values are start and end of the exon.

**Figure 2:**
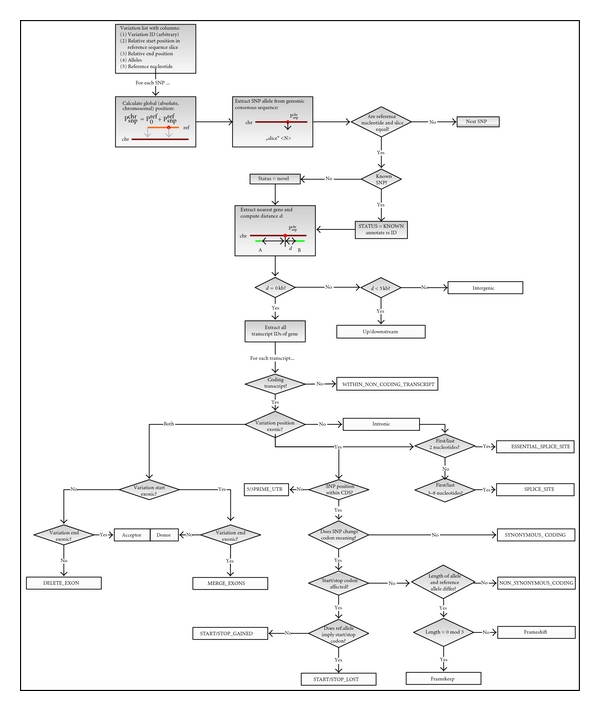
The workflow of NovelSNPer. Each variation from a list is checked if it is a previously known variation or a novel variation, then the variation is attributed to one or several out of twenty-one functional classes.

Afterwards, the genetic code is loaded in the cache. Then the following steps are performed for each variation in the input file (see Figure 2 for the flowchart).

- The chromosomal position ChromPos (“global position”) of the variation under investigation is calculated as sum of the relative position of the variation and the start position of the reference sequence with respect to the chromosome.
- A sequence object (“slice”) of length one nucleotide is generated from the chromosomal sequence at the global position of the variation.

(iii) The reference nucleotide from the input is compared with the nucleotide obtained from the chromosomal sequence. If these do not coincide this variation is not processed and all output variables are set to NA, but it is kept in the output for reference purposes.
(iv) The alternative allele sequence is compared with the reference sequence. Depending on this comparison, the variation is classified as single nucleotide polymorphism (SNP), multinucleotide polymorphism (MNP), inversion or indel.
  - *SNP*: alternative allele and reference allele are exactly one base pair long.
  - *Inversion*: the alternative allele is the inversion of the reference allele and it is longer than one base pair.
  - *MNP*: alternative allele and reference allele have the same length, but it is neither an SNP nor an inversion. (for visualization: some SNPs which lay together.)
  - *Indel*: alternative allele and reference allele have different length.
(v) If the variation is an indel, the sequence of the variation is compared with 13 bp of the flanking sequence to determine, if it is a potential transposon [24].
(vi) The nearest gene (NearestGene) and the distance (Distance) to it in base pairs are determined. The distance is given as negative number if the nearest gene is found upstream of the variation and as positive number if it is downstream.
(vii) If the sequence slice harbours a variation the variation to be examined is recognized as known variation, otherwise the variation is considered as novel. The variable Status is set to KNOWN or NOVEL, respectively. If an input variation has the same coordinates as a known variation, but its alleles do not coincide with the alleles in the database, its status is set to ALTERNATIVE.
(viii) If the distance to the nearest gene exceeds 5 kb, the variation is classified as INTERGENIC, if the distance is greater than 0 but less or equal 5 kb, the variation is classified as UPSTREAM or DOWNSTREAM, respectively. Upstream regions of this extent usually harbour the promoters [25]. The nearest gene and the distance to it are given for both novel and known variations. If the variation is within a gene (distance = 0) the following steps are performed for all its transcripts since the effect of a variation can be dependent on its context. First, it is decided if the variation is located in an exon, an intron, or if it overlaps both. In the second case the variation is classified as INTRONIC. If the variation is in an exon, it is determined if it is in the noncoding or in the coding part of an exon. If it is in the noncoding part of an exon, the variation is classified as 5PRIME_UTR and 3PRIME_UTR, depending on whether it is located before the start codon or after the stop codon. If it is in the coding part of an exon, it is determined if the variation changes the length of the protein or not. If it changes the length, it is FRAMEKEEP, respectively, FRAMESHIFT. If the variation does not change the length of the protein, it is checked if the variation entails an amino acid exchange in the resulting protein sequence or not; the variation is classified as NON_SYNONYMOUS_CODING or SYNONYMOUS_CODING, respectively. If one of the SNP alleles is part of a stop codon the SNP is considered as STOP_LOST or STOP_GAINED depending on whether the reference allele or the alternative allele constitutes the stop codon. If an SNP is located in the first two or in the last two nucleotides of an intron it is classified as ESSENTIAL_SPLICE_SITE. If an SNP is within the first three to eight or last three to eight nucleotides of an intron or within the first or last three nucleotides of an exon, it is classified as SPLICE_SITE. If an SNP is located within a mature miRNA or within a non-coding gene, it is classified as WITHIN_NON_CODING_TRANSCRIPT.
We label an SNP as START_LOST if it is a nonsynonymous SNP in a start codon. Finally, we label an SNP as POTENTIAL_START_GAINED if the SNP is located in the 5' UTR of a protein-coding gene or within the exon of a noncoding transcript and if the variation results in the creation of an ATG start codon.
(ix) Each variation in the coding part of an exon is checked if the corresponding amino acid is part of a Pfam protein domain [26].
(x) The difference of expected and observed evolutionary conservation across 34 mammalian species is calculated for each variation [27].

### 2.4. Runtime

NovelSNPer processes on average 10,000 variations in a continuous 35 Mbp region in 2 minutes, if it is connected to Ensembl. If NovelSNPer works offline, the annotation takes a minute. The analysis of 2 million variations obtained by resequencing a bovine genome [28] took 6 hours without calculation of conservation scores. Including the calculation of conservation score the analysis took 14 hours. The run was made on a 2,2 Ghz AMD Processor with 2 GB RAM.

### 2.5. New Functional Classes

#### 2.5.1. Transposon

 Transposons are parts of the DNA, which have the ability to copy or move themselves. Thus the corresponding variation can differ within the same individual. The identification of transposons is useful to estimate, if the sequenced variation exists on the whole individual or if the sequence is only valid for the sequenced cell.

Also the pattern of the sequence shows if the variation is an inserted transposon or if the variation occurs because of a transposon which already shifted away.

#### 2.5.2. Start Gained/Start Lost

 To estimate how strong a variation in genotype affects the phenotype, it is helpful to look at the proteome. A variation, which leads to a change in the protein, can have a profound effect on the phenotype. There exist variations which alter the initiation code and thus lead to a possible suppression of the protein [29]. Also an altering of the protein due to a variation in the translation start is well discussed [30–32]. Hence, in addition to the traditional variations, NovelSNPer also searches for changes in the start codon and determines if a variation in the 5′-UTR leads to a new potential start codon.

#### 2.5.3. Subclasses of Complex Indels

 Deletions can overlap the coding region and the noncoding region. Depending on their location and length they can have different functions. They can delete a whole exon, merge two exons by deleting the intron, delete the acceptor, or delete the donor. If the variation deletes a whole exon or merges two exons, it is possible to determine the alternative protein exactly. If the deletion only deletes the splice site it is not possible to calculate the alternative protein, because the emerging splice pattern is unknown.

**Figure 3:**
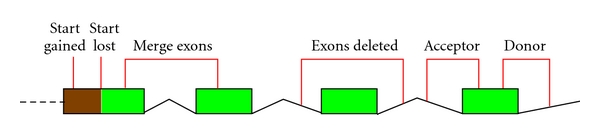
New functional classes. Visualization of the new functional classes.

This is one reason why it is essential to subclassify the complex indels. NovelSNPer uses the self-describing classes MERGE_EXONS, DELETE_EXONS, ACCEPTOR, and DONOR (Figure 3).

### 2.6. Evolutionary Genesis

As an option, NovelSNPer can analyse if a given indel is a duplication of the adjacent sequence, an inversion, or a shifting element. Although it does not reveal anything about the function of the indel, it gives a hint of the evolutionary genesis of the indel and the mechanism how this indel has derived.

Also it calculates the repeat-length of an indel. A difference in the length of the indel and the period-length of the indel indicates that there were multiple mutations which led to this indel.

### 2.7. Genetic Code

NovelSNPer loads the standard genetic code at startup per default. But it is optionally possible to use the genetic code of prokaryotes, yeasts, mycoplasma, or mitochondria (as given in [33–35]).

## 3. Results

### 3.1. Reliability

 We wrote the Perl script NovelSNPer.pl that allows for easy and fast analysis of variation lists produced in a next-generation sequencing project. This script first determines if a variation identified in the sequence data corresponds to an already known variation that is stored in the Ensembl database or if it is a previously unknown variation. Unknown variations are classified into one or several out of a total of 21 functional classes. The functional class of an variation permits the scientist to judge the consequences that a variation has for gene expression or protein conformation. Variations are furthermore assigned to a gene if they are located within that gene or the distance to the nearest gene is given. Variations can be assigned to two genes if these overlap on the two strands of the genome.

To test our tool, we compared the annotation attributed by NovelSNPer with that given by Ensembl (version 61). For this purpose, we tested all 30 million human variations represented in Ensembl. The classes POTENTIAL_START_GAINED and START_LOST could not be tested because they were uniquely used by NovelSNPer. The variations with the classes MERGE_EXONS, DELETE_EXONS, ACCEPTOR, and DONOR were considered as correct if the variation is a COMPLEX_INDEL in Ensembl.

Altogether, in 99.8% of all variations there was no difference between Ensembl and NovelSNPer. Only 0.2% of all variations are classified into different classes. 60% of these variations are classified as INTERGENIC by Ensembl while NovelSNPer found a gene containing the variation (e.g., rs68103157 is considered as INTERGENIC by Ensembl while it is considered as INTRONIC for the gene SOAT1 by NovelSNPer); 10% of the differently-classified variations are classified as FRAMESHIFT by Ensembl while NovelSNPer classified them as SYNONYMOUS_CODING or NON_SYNONYMOUS_CODING. An analysis of these variations revealed that the length of the alternative allele and the length of the reference allele are the same, but greater than 1 (e.g., the alternative allele and the reference allele of rs71585892 has a length of 5 bases).

An SNP was considered as classified in agreement with Ensembl if the classes coincided. In cases where NovelSNPer assigned more than one class, the class given by Ensembl had to be among the classes that were assigned by NovelSNPer in order to be considered as agreement.

**Figure 4:**
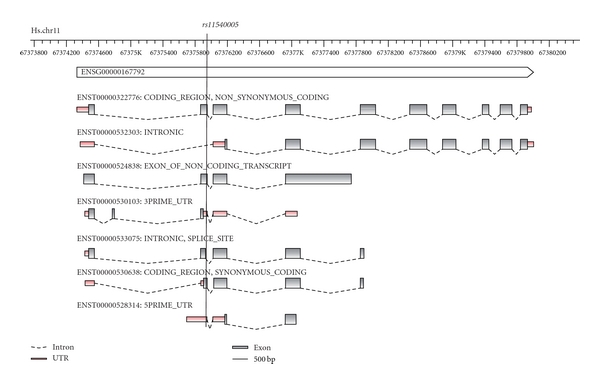
Example of a multi-class SNP. SNP *rs11540005* can be assigned to seven functional classes in the human *NDUFV1* gene. The *NDUFV1* gene seems to be related with dilated cardiomyopathy [36]. Shown are seven transcripts from the diverse transcriptional landscape at this genomic site. Graphical visualization was done with *fancyGene* [37].

Interestingly, it is not an exception that an SNP is assigned to more than one functional class, but about half of the SNPs belong to more than one functional class. One SNP (*rs11540005*) belonged to seven classes. This can be understood considering the fact that one gene is typically made up of several transcripts or splice variants, and the role of an SNP can be dependent on the transcript variant (Figure 4). For example, the reading frame can vary between two transcripts, and, hence, one and the same SNP can be SYNONYMOUS_CODING in one transcript and NON_SYNONYMOUS_CODING in the other. Even within one transcript, an SNP can take on more than one functional class, for example, an SNP can be simultaneously of type SPLICE_SITE and of type 3PRIME_UTR. Last, but not least, there are rare cases where two genes overlap, and an SNP at such a locus occupies two different roles in these two genes.

### 3.2. New Classes of Variants

Since we were not aware of any systematic genome wide analysis of start gained and start lost SNPs, we were interested to study the abundance of such cases. We found that 1,418 of 23,621 human protein coding genes (6.0%) dispose of at least one alternative start gained SNP and 218 (1.0%) of at least one start lost SNP. We furthermore identified 5,585 genes of a total of 49,509 (protein-coding and nonprotein-coding) genes (11.3%) that have at least one non-coding transcript, which harbours at least one potential start gained SNP. The lists of SNPs are available at our website (http://www2.hu-berlin.de/wikizbnutztier/software/NovelSNPer/).

### 3.3. Indels with Ambiguous Identification

 In a test run we checked all 4 million insertions and deletions of the human genome represented in Ensembl. We found over 20,000 indels with ambiguous annotation. This means several different indels in the Ensembl database are the same indel in reality. A list of some of these multiple indels is available as Additional File 4. As an example the deletions *rs71918324, rs71702364, rs5796236, rs35226411, rs72397401, rs35471040*, and* rs61916428* on chromosome 12 between position 6551619 and 6551638 represent one and the same indel. The details of this indel can be seen in Table 5. An ambiguous deletion of length six and its 6 bp-sequence is shown in Table 6(a). An ambiguous deletion which is 3 bp long and has five different identifiers is shown in Table 6(b).

It is not sufficient to compare only the position of indels. It is not even certain to have two different indels, if the alleles are different. To decide whether two indels are the same or not the sequence between this two alleles has to be considered. Even though the percentage of multiple annotated indels is very low NovelSNPer offers an effective way to identify these misleading annotations.

### 3.4. Bovine Datasets

In a second run NovelSNPer annotated 2.3 million variations of the bovine genome, which were published in [28]. The variation data-set consists of 2,307,509 SNPs and 683 indels. 639 of these indels are a duplication of the previous nucleotide and 44 indels are an insertion of a new nucleotides. In Table 7 the distribution of the variations across genomic regions is shown. The distribution into functional classes of all variations in coding regions is shown in Table 8.

## 4. Discussion

 Whole genome resequencing experiments are performed to systematically identify genomic variations. For example, the complete genome of a single * bos taurus* animal was sequenced to identify millions of previously unknown cattle SNPs [28]. In another work, artificial mutations that are responsible for phenotypes in * caenorhabditis elegans* could be identified thanks to whole genome sequencing [38]. Distilling the huge quantity of information into meaningful lists of SNPs is a multi-step bioinformatics process. NovelSNPer is an easy to use tool that helps scientists with the analysis of next-generation sequencing data. Lengthy lists of SNPs from next-generation resequencing projects are efficiently assessed and annotated with the most important SNP features. Of outmost interest is the functional class of an SNP. SNPs involving stop gains (nonsense mutations) should in most cases mediate severe impairment of a protein's functioning. Non-synonymous SNPs can also entail a modification of a protein's conformation depending on how dissimilar the exchanged amino acids are. Whereas these two classes of SNPs can have a more or less direct effect on a protein (see, e.g., [39]), SNPs in untranslated regions (UTRs), introns, and up- and downstream regions have the potential to alter the binding behaviour of transcription factors or splice factors and, thus, to alter gene expression, indirectly.

Finally, variations in the immediate neighbourhood of exon-intron boundaries could influence the splicing process of a transcript. One important type of genomic variation are frame shifts, that is, disruptions of the reading frame. This variation is mediated by indels of one nucleotide or of a stretch of nucleotides whose length is not a multiple of three into the coding part of a gene.

Two more variation features permit life scientists to assess the severeness of the consequences a variation can have: (a) the protein domain [26] that is affected by an indel or a nonsynonymous SNP and (b) the difference between observed and expected conservation score [27]. A great difference between observed and expected conservation score suggests that there is negative selection pressure more than expected to maintain the nucleotide at that position. A variation at such a position should therefore be likely to have negative consequences for the phenotype. Protein domains can be considered as protein building blocks that occur in various proteins within a species and across species. They are more conserved than those parts of the protein that are not organized in protein domains. A non-synonymous SNP that leads to the exchange of an amino acid that is part of a protein domain should therefore have more severe consequences for the phenotype than a non-synonymous SNP that affects an amino acid outside a protein domain.

NovelSNPer is one of the few tools that explicitly classifies variations as POTENTIAL_START_GAINED or START_LOST if they mediate between a start codon and a non-start codon in the assembly and the reference sequence. We found such a classification only in the Mouse SNP database by the Center for Genome Dynamics of the Jackson Laboratory http://cgd.jax.org/cgdsnpdb/.

A reason why other tools or databases are obviously reluctant to use these two classes might be that a start codon alone is not sufficient to predict the translation start for sure. A variation which is classified as POTENTIAL_START_GAINED is a * potential* new start codon that competes with the previously existing start codon (if this has remained unaltered). In contrast, a START_LOST variation can be considered as unambiguous signal that the translation start is shifted as compared to the wild type. Alternative translation start sites were discussed, for example, in [30, 31]. The figures that we presented in Section 3 suggest that the protein synthesis of quite a substantial number of genes can be altered by POTENTIAL_START_GAINED or START_LOST variations. One percent of the genes appear to be susceptible to protein synthesis suppression altogether.

By querying the Ensembl database online, NovelSNPer does not need to be maintained regularly. Its great value is furthermore that resequencing data from all species whose genome sequence is available in Ensembl can be processed. By using NovelSNPer offline it is possible to annotate variations for all species as long as the genome-structure of the transcripts is available as BED file or GFF file [19, 20].

NovelSNPer completes the functionality of existing tools in many ways: variation calls are checked against the reference sequence to guarantee the correctness of the results, the usage of relative SNP positions is possible, the novel functional classes POTENTIAL_START_GAINED and START_LOST are introduced, heterozygous variation calls can be treated, there is no limit in the number of variations to be processed, and information about the degree of conservation and protein domains is presented. SNPs and indels can be processed alike.

## 5. Conclusions

To make full usage of the data that are generated in next-generation-sequencing experiments, the lists of called variations must be efficiently screened. NovelSNPer is a fast tool that exploits the annotations about genes and genomes from the Ensembl database to classify each called variation as novel or as previously existing. Each variation is classified into one or more of twenty-one functional variation classes. Two of these classes, START_LOST and POTENTIAL_START_GAINED, were thus far rarely included in SNP analysis programs. However, we showed that these two types of functional classes are quite frequent and could play a significant role in protein synthesis. The great number of species that can be analysed, the structured and detailed outputs, and the integration of new features like additional variation classes or the calculation of the conservation score for each variation make NovelSNPer a versatile analysis tool. Also the possibility to predict the effect of variations on a newly discovered transcript which is not in a public database is very useful. We thus anticipate a wide range of applications in the biological, medical and agricultural sciences.

## 6. Availability and Requirements

NovelSNPer is implemented in Perl using Bioperl and Ensembl's Perl APIs. The latest version of the program can be downloaded free of charge from http://www2.hu-berlin.de/wikizbnutztier/software/NovelSNPer/. The source code is also available at the website.

NovelSNPer runs under Linux and Windows and needs at least 512 MB RAM. MySQL, Perl, BioPerl and the API modules ensembl, ensembl-compara, and ensembl-variation (application protocol interfaces http://www.ensembl.org/info/docs/api/index.html) have to be installed locally. Alternatively, the web interface can be used.

## Figures and Tables

**Table 1:** List of variations. Name and description of all variation classes used in NovelSNPer. A cross (X) indicates that a variation class can be assigned to an SNP, MNP, or InDel, respectively.

Name	SNP	MNP	InDel	Description
Intergenic	X	X	X	More than 5000 bp away from any gene
Upstream	X	X	X	Upstream the nearest gene and this gene is closer than 5000 bp
Downstream	X	X	X	Downstream the nearest gene and this gene is closer than 5000 bp
Within noncoding transcript	X	X	X	In the exon or intron of a noncoding gene
Intronic	X	X	X	In the intron of a coding gene
5′ UTR	X	X	X	In the exonic 5′ UTR
3′ UTR	X	X	X	In the exonic 3′ UTR
Synonymous coding	X	X		Variation in a coding region without changing the amino acid
Nonsynonymous coding	X	X		Variation in a coding region with changing a single amino acid
Framekeep			X	Insertion or deletion of some amino acids
Frameshift			X	Changing the reading frame in coding region
Stop gained	X	X	X	Generates a new stop codon in the coding region
Stop lost	X	X	X	Deletion of an existing stop codon at the end of coding region
Potential start gained	X	X	X	Generates a potential start codon
				in 5′ UTR or in an exon of noncoding transcript
Start lost	X	X	X	Deletion of an existing start codon at the beginning of coding region
Delete exon			X	Deletion of a whole exon
Merge exon			X	Deletion of a whole intron
Acceptor		X	X	Deletion of the exonic and intronic splice site upstream of an exon
Donor		X	X	Deletion of the exonic and intronic splice site downstream of an exon
Splice site	X	X	X	Variation near the exon-intron boundary
Essential splice site	X	X	X	Intronic variation within 3 bp range of an exon-intron boundary

**Table 2:** Example input file. An input file Input.txt containing seven variations.

Name	Chromosome	RelStart	RelEnd	AltAllele	RefAllele
var1	12	12483171	12483171	C	G
var2	12	12483200	12483200	G	T
var3	12	12506184	12506184	T	—
var4	12	12527105	12527108	GATT	TTAG
var5	12	12550471	12550471	A/G	T
var6	12	12588583	12588583	A	C
var7	12	12588586	12588587	—	CA

**Table 3:** Example basic output file. NovelSNPer generates the output file Output_basic.txt with one line per variation.

Name	Chr	Start	End	Region	Type	FuncClass	NearestGene	Status	rsID
var1	12	12483171	12483171	CODING_REGION	SNP	SYNONYMOUS_CODING	MANSC1	KNOWN	rs113135329
var2	12	12483200	12483200	CODING_REGION	SNP	NON_SYNONYMOUS...	MANSC1	NOVEL	NA
var3	12	12506184	12506184	DOWNSTREAM	INDEL	NONE	LOH12CR2	KNOWN	rs113229925, rs11313889
var4	12	12527105	12527108	INTRONIC	INVERSION	NONE	LOH12CR1	NOVEL	NA
var5	12	12550471	12550471	INTRONIC	SNP	NONE	LOH12CR1	NOVEL	NA
var6	12	12588583	12588583	CODING_REGION	SNP	NON_SYNONYMOUS...	LOH12CR1	KNOWN	rs76204637
var7	12	12588586	12588587	CODING_REGION	INDEL	FRAMESHIFT	LOH12CR1	NOVEL	NA

**Table 4:** Example detailed output file. NovelSNPer generates the output file Output_detailed.txt with one line per variation per transcript.

Name	Chr	Start	End	Allele	RefAllele	Codon	AA	refAA	Class	Transcript	Strand
var1	12	12483171	12483171	C	G	GCS	Ala	Ala	SYNONYMOUS_CODING	ENST00000355566	− 1
var1	12	12483171	12483171	C	G	GCS	Ala	Ala	SYNONYMOUS_CODING	ENST00000396349	− 1
var2	12	12483200	12483200	G	T	MAA	Gln	Lys	NON_SYNONYMOUS_CODING	ENST00000355566	− 1
var2	12	12483200	12483200	G	T	MAA	Gln	Lys	NON_SYNONYMOUS_CODING	ENST00000396349	− 1
var3	12	12506184	12506184	T	—	NA	NA	NA	DOWNSTREAM	LOH12CR2	− 1
var4	12	12527105	12527108	GATT	TTAG	NA	NA	NA	INTRONIC	ENST00000298571	1
var4	12	12527105	12527108	GATT	TTAG	NA	NA	NA	INTRONIC	ENST00000314565	1
var5	12	12550471	12550471	A/G	T	NA	NA	NA	INTRONIC	ENST00000298571	1
var5	12	12550471	12550471	A/G	T	NA	NA	NA	INTRONIC	ENST00000314565	1
var6	12	12588583	12588583	A	C	TMC	Tyr	Ser	NON_SYNONYMOUS_CODING	ENST00000298571	1
var6	12	12588583	12588583	A	C	TMC	Tyr	Ser	NON_SYNONYMOUS_CODING	ENST00000314565	1
var7	12	12588586	12588587	—	CA	C[-/CA]A	Cis	ProThr	FRAMESHIFT	ENST00000298571	1
var7	12	12588586	12588587	—	CA	C[-/CA]A	Cis	ProThr	FRAMESHIFT	ENST00000314565	1

**Table 5:** Ambiguous short indels. Multiple deletion annotation on human chromosome 12. The deleted nucleotide A is underlined in the reference sequence. In the new sequence a gap at the position of the deletion is shown.

Name	Position	refAllele	Allele	Reference sequence	Alternative sequence
rs71918324	6551619	A	—	TCTCAAAAAAAAAAAAAAAAAAAAGAAC	TCTC AAAAAAAAAAAAAAAAAAAGAAC
rs71702364	6551627	A	—	TCTCAAAAAAAAAAAAAAAAAAAAGAAC	TCTCAAAAAAAA AAAAAAAAAAAGAAC
rs5796236	6551628	A	—	TCTCAAAAAAAAAAAAAAAAAAAAGAAC	TCTCAAAAAAAAA AAAAAAAAAAGAAC
rs35226411	6551629	A	—	TCTCAAAAAAAAAAAAAAAAAAAAGAAC	TCTCAAAAAAAAAA AAAAAAAAAGAAC
rs72397401	6551630	A	—	TCTCAAAAAAAAAAAAAAAAAAAAGAAC	TCTCAAAAAAAAAAA AAAAAAAAGAAC
rs35471040	6551638	A	—	TCTCAAAAAAAAAAAAAAAAAAAAGAAC	TCTCAAAAAAAAAAAAAAAAAAA GAAC

**Table:** (a) Ambiguous long indels. Multiple deletion notation on human chromosome 14. The nucleotides of the deletion are underlined in the reference sequence. In the new sequence a gap at the position of the deletion is shown.

Name	Start	End	Alleles	Reference sequence	Alternative sequence
rs3841049	21560753	21560758	GAGGCT/-	GTGGAGGCTGAGGCTGAGGCTGAGGCGG	GTG GAGGCTGAGGCTGAGGCGG
rs71814523	21560759	21560764	GAGGCT/-	GTGGAGGCTGAGGCTGAGGCTGAGGCGG	GTGGAGGCT GAGGCTGAGGCGG
rs72383174	21560762	21560767	GCTGAG/-	GTGGAGGCTGAGGCTGAGGCTGAGGCGG	GTGGAGGCTGAG GCTGAGGCGG
rs7179484	21560764	21560759	GAGGCT/-	GTGGAGGCTGAGGCTGAGGCTGAGGCGG	GTGGAGGCTGAGGC TGAGGCGG

**Table:** (b) Ambiguous long indels. Multiple deletion notation on human chromosome 19. The nucleotides of the deletion are underlined in the reference sequence. In the new sequence a gap at the position of the deletion is shown.

Name	Start	End	Alleles	Reference Sequence	Alternative Sequence
rs3840928	30500119	30500121	TGA/-	AGTGATGATGATGATGATGATGATGATGACG	AG TGATGATGATGATGATGATGATGACG
rs71645759	30500127	30500129	ATG/-	AGTGATGATGATGATGATGATGATGATGACG	AGTGATGATG ATGATGATGATGATGACG
rs67383412	30500129	30500131	GAT/-	AGTGATGATGATGATGATGATGATGATGACG	AGTGATGATGAT GATGATGATGATGACG
rs10559374	30500130	30500132	ATG/-	AGTGATGATGATGATGATGATGATGATGACG	AGTGATGATGATG ATGATGATGATGACG
rs58360763	30500143	30500145	TGA/-	AGTGATGATGATGATGATGATGATGATGACG	AGTGATGATGATGATGATGATGATGA CG

**Table 7:** Bovine variations in various genomic regions. Distribution of variations found in the bovine genome in an NGS experiment. The sum of the percentage is higher than 100%, because some variations are in several transcripts and can therefore be allocated to multiple regions.

Region	Number	Percentage
INTERGENIC	1,485,588	64.36%
INTRONIC	647,129	28.04%
UPSTREAM	73,996	3.21%
DOWNSTREAM	73,512	3.18%
CODING_REGION	20,856	0.90%
3PRIME_UTR	6,019	0.26%
SPLICE_SITE	2,364	0.10%
5PRIME_UTR	1,303	0.06%
WITHIN_NONCODING_TRANSCRIPT	786	0.03%
ESSENTIAL_SPLICE_SITE	133	0.01%

**Table 8:** Exonic functional classes of bovine variations. Distribution of variations found in the coding region of the bovine genome. The sum of the percentage is higher than 100%, because some variations are in several transcripts and can therefore be classified into several functional classes.

Functional class	Number	Percentage
SYNONYMOUS_CODING	12,327	59.11%
NON_SYNONYMOUS_CODING	8,464	40.58%
STOP_GAINED	83	0.40%
POTENTIAL_START_GAINED	48	0.23%
FRAMESHIFT	11	0.05%
STOP_LOST	9	0.04%
START_LOST	5	0.02%
